# Moral Injury in Mental Health Nursing—A Qualitative Descriptive Study in Switzerland

**DOI:** 10.1111/inm.70099

**Published:** 2025-07-24

**Authors:** Célina Heitzmann, Veronika Waldboth, Mirjam Mezger

**Affiliations:** ^1^ Zurich University of Applied Sciences ZHAW Winterthur Switzerland; ^2^ University Department of Geriatric Medicine FELIX PLATTER Basel Switzerland

**Keywords:** ethics nursing, mental health nursing, moral injury, qualitative research, trauma and stressor related disorders

## Abstract

Moral injury (MI) is the damage done to one's conscience or moral compass when one perpetrates, witnesses, or fails to prevent acts that transgress one's own moral beliefs. There are numerous associations between MI and various mental health outcomes, including burnout, depression, anxiety, sleep disorders, and increased job turnover. However, there has been no research or official study investigating MI in mental health nurses (MHNs) in Switzerland. This study aimed to examine and describe the spectrum and impact of MI in Swiss MHN. Demographic data and descriptions of MI in mental health nursing were collected from 19 problem‐focused interviews between September and November 2023. The data were analysed descriptively and using qualitative content analysis strategies, respectively. Participants highlighted specific potentially morally injurious events (PMIEs) such as coercive measures, power plays, and sanctioning behaviour prevalent in mental health nursing. As they caused violations of moral values, with intense emotional responses ranging from anger to guilt, shame, helplessness, and powerlessness, MIs could be identified. They resulted in long‐term consequences such as job changes, sleep disturbances, anxiety, panic attacks, sensory crises, and substance abuse. The results emphasise the impact of MI on the well‐being and practice of MHN in Switzerland. Participants perceived MI as expressing intense emotions and dissatisfaction, challenging their moral principles in the context of their daily work. Participants confronted with MI reported increased risks for negative health outcomes. The identification of causes of MI emphasises the need for targeted interventions in the psychiatric setting.

## 1 Background

Moral Injury (MI) research focuses on morally challenging situations and the potential impact of such experiences on individuals (Grimell and Nilsson 2020). Research on MI has its origins in veterans' research, with the term first emerging in the 1990s in the context of war psychology (Litz et al. 2009). Particularly during the COVID‐19 pandemic, there has been an increase in research on MI, especially in acute care settings, with a particular focus on nurses working on the frontline (Čartolovni et al. 2021; Maguen and Griffin 2022). Moral distress is the mental unease experienced when one recognises the ethically correct course but is barred from executing it. It is typically situational and often resolves once the constraint is lifted (Epstein and Hamric 2009). MI stems from perceived ethical transgressions and correlates with profound, enduring emotional, psychological, or spiritual distress. Not every case of moral distress results in MI, but repeated exposure to challenging ethical situations may raise the likelihood of this happening (Litz et al. 2009). MI can be defined as psychological distress arising from potentially morally injurious events (PMIEs) (Greenberg et al. 2020). According to Litz and Kerig (2019), committing, omitting, preventing, witnessing, or learning about actions that injure deeply rooted moral beliefs and expectations are PMIEs that can have long‐term impacts on psychological, emotional, social, and spiritual health. A PMIE leads to MI when an individual perceives that a significant moral value has been injured by their own or others' actions and manifests at an emotional, psychological, and behavioural level. This perception reflects the consequences of MI rather than the conditions under which a PMIE becomes morally injurious (Farnsworth et al. 2017). Litz and Kerig (2019) assume that the risk of MI increases with the duration and frequency of exposure to moral stressors, moral dilemmas, and morally challenging situations. MI can be understood as an extreme along a continuum, caused by moral stressors, morally challenging situations, moral dilemmas, and PMIEs. PMIEs are also situated at the end of this continuum as they are defined as moral transgressions and considered a necessary but not sole condition for MI (Litz and Kerig 2019; Rowlands 2021; Williamson et al. 2018).

PMIEs are distinguished between betrayal‐based and self‐based. Self‐based PMIEs involve intentional or unintentional actions committed by one's person that injure one's moral values, while betrayal‐based PMIEs involve immoral actions by a trusted person (Jordan et al. 2017). MI is depicted as an extreme psychological distress emerging within this interplay, where PMIEs play a pivotal role within the broader context of moral stressors. Most descriptions of MI symptoms include feelings of shame, guilt, disgust, loss of self‐trust, and secondary symptoms such as depression, anxiety, anger, and social problems (Jinkerson 2016; Stovall et al. 2020; Williamson et al. 2020). The immediate risk of MI is that healthcare workers (HCW) no longer verbalise their dilemmas and may withdraw from discussions about ethically challenging decisions or actions in practice, feeling internally powerless to resolve the outward moral conflict (Welborn 2019). Jovarauskaite et al. (2022) examined the associations between MI and post‐traumatic stress disorder (PTSD) in HCW. Among the 206 participants, 20% reported trauma‐related disorders, while 61.2% were exposed to PMIEs. The HCW interviewed in their study described PMIEs as physical abuse by patients, witnessing intentional harm to patients by co‐workers, conflicts with patients or co‐workers, unintentional errors, unfair treatment regarding working conditions, and emotional abuse. Bowers et al. (2006) report severe adverse incidents, such as suicides, suicide attempts, severe injuries or escape of high‐risk patients in acute psychiatric wards. These incidents were traumatic for staff members and were associated with feelings of guilt and fear. In the scientific commentary by Looi et al. (2022) on MI among psychiatrists and trainees during the COVID pandemic, such serious adverse incidents in psychiatry are considered PMIEs by psychiatrists and may lead to MI and burnout. However, the specific PMIEs to which MHN are exposed were not considered. Jansen et al. (2022) showed that MHNs are exposed to moral distress due to manifold ethical dilemmas, conflicting demands, and their proximity to the suffering of their patients, including coercive treatments such as forced medication and measures that restrict freedom. This exposure often stems from uncertainty about ethical boundaries, particularly about when to use coercive measures, leading to an increased risk of threats and violence (Jansen et al. 2022). Therefore, MHNs are at risk of experiencing MI.

Despite growing awareness of MI, limited knowledge exists on how MHNs experience MI, the triggers they identify, their perception of MI, and its impact on their well‐being and nursing practice. This qualitative, descriptive study investigates MHN experiences with MI in Switzerland, examining its implications for well‐being and practice. Specifically, it explores how MHNs perceive and encounter MI in psychiatric settings, its effects on well‐being and professional practice, and potential triggers. The study aims to offer insights for mental health nursing and inform targeted interventions, generating recommendations applicable to nursing practice globally.

## 2 Methods

### 2.1 Study Design

A qualitative descriptive research approach is adopted for this study. Abiding by the principles of social constructionism, the study aims to explore and understand the experiences of MI from the subjective perspective of the individuals affected (Moon and Blackman 2014). This approach seeks to provide straightforward descriptions of experiences and perceptions related to the phenomenon of MI (Sandelowski 2000). Reporting of this study adheres to the Standards for Reporting Qualitative Research (SRQR) and involves the analysis of demographic data and problem‐centred interviews concerning MI experienced by participants (O'Brien et al. 2014).

### 2.2 Study Setting and Participants

This study was conducted in German‐speaking Switzerland. The data were collected between August and November 2023 and involved in‐person interviews at locations chosen by the participants. Eligible participants included MHNs with at least one year of experience in mental health nursing. Recruitment was carried out via email to personal contacts in two selected psychiatric clinics, targeting gatekeepers for study population access. Additionally, gatekeepers uploaded an intranet announcement with an attached flyer, allowing potential participants to contact the researchers directly. The purposive sample consisted of 19 participants, with inclusion criteria being the completion of basic nursing training and employment in a psychiatric setting.

### 2.3 Ethical Considerations

This study is not subject to the Swiss Human Research Act and has been authorised by the Zurich University of Applied Sciences (ZHAW) Ethics Review Board (EA‐ZHAW‐STA‐003‐G). It was conducted in accordance with the Declaration of Helsinki (World Medical Association 2013). Data collection commenced only after obtaining the necessary ethical approval, ensuring compliance with all relevant ethical considerations. Participation required written informed consent, understanding of the voluntary nature of participation, and the option to terminate interviews or object to audio recording use if desired; all data were anonymised. A distress protocol was implemented to address potential emotional distress during interviews, ensuring participants' well‐being (Whitney and Evered 2022). Although none of the participants needed formal assistance during or following the interviews, the researcher stayed alert for any signs of discomfort, provided breaks when necessary, and emphasised that they could pause or end the interview at any moment.

### 2.4 Data Collection

To capture the experiences and subjective perceptions of MI among MHN, problem‐centred interviews were conducted, with an emphasis on a specific problem area (Döringer 2020). A standardised interview guide was developed using a set of questions, which was created using existing literature and the research questions (Creswell and Creswell 2022). To obtain systematically analysable data, the interviews were recorded as audio files and subsequently verbatim transcribed and anonymised (Dresing and Pehl 2020). Additionally, demographic data, including gender, age, educational level, current institution/ward, employment percentage, and years working in mental health, were collected to describe the study population.

### 2.5 Data Analysis

Sociodemographic information was analysed descriptively using Excel. The collected qualitative data were analysed using the content‐structuring qualitative content analysis according to Kuckartz (2019), employing thematic categories. This approach aims to summarise the information content of the data (Kuckartz 2019).

In the initial step, key passages were highlighted to provide an overview of the data material. Subsequently, thematic main categories were developed based on the research questions, existing concepts on the phenomenon of MI, and data material. In the following step, the entire dataset was coded with the established main categories. Further coding involved the inductive development of additional main categories from the data material. The entire dataset was systematically worked through sequentially, and each segment was assigned to its respective thematic categories. Inductively, the analysis of the data material resulted in the identification of subcategories, allowing for a detailed differentiation of the existing category system. The entire dataset was then coded using the developed and differentiated category system. In the final step, all main categories were reviewed to identify connections between the categories. This aimed to highlight any associations or interactions between the identified thematic areas. Finally, the results of the analysis, including detailed descriptions of the identified categories, were summarised and documented in a report (Kuckartz 2019). The coding process was supported through regular discussions with the supervising researcher and within a peer research colloquium. While no inter‐coder agreement or member checking was conducted, this collaborative exchange helped ensure consistency and transparency in the analysis.

## 3 Results

Of the 19 participants, 14 were women, and the overall average age was 42 years (Table 1). They had an average of 14.4 years of mental health work experience, and the interviews lasted on average 40 min.

**TABLE 1:** Sample descriptors *n* = 19.

Descriptor	Category	*n* =	$\bar{x}$ =
Gender	Female	14	
	Male	5	
	Other	0	
Age	21–29	5	42
	30–39	2	
	40–49	6	
	50–59	6	
	> 60	0	
Educational level	Federal Diploma of Vocational Education and Training	1	
	Advanced Federal Diploma of Higher Education	12	
	Bachelor in Nursing	6	
Setting	Acute inpatient setting	7	
	Psychotherapeutic setting	8	
	Forensic Psychiatry	2	
	Outpatient clinic/Day clinic	2	
Years working in mental health	1–9	7	14.4
	10–19	4	
	20–29	7	
	> 29	1	

**FIGURE 1:**
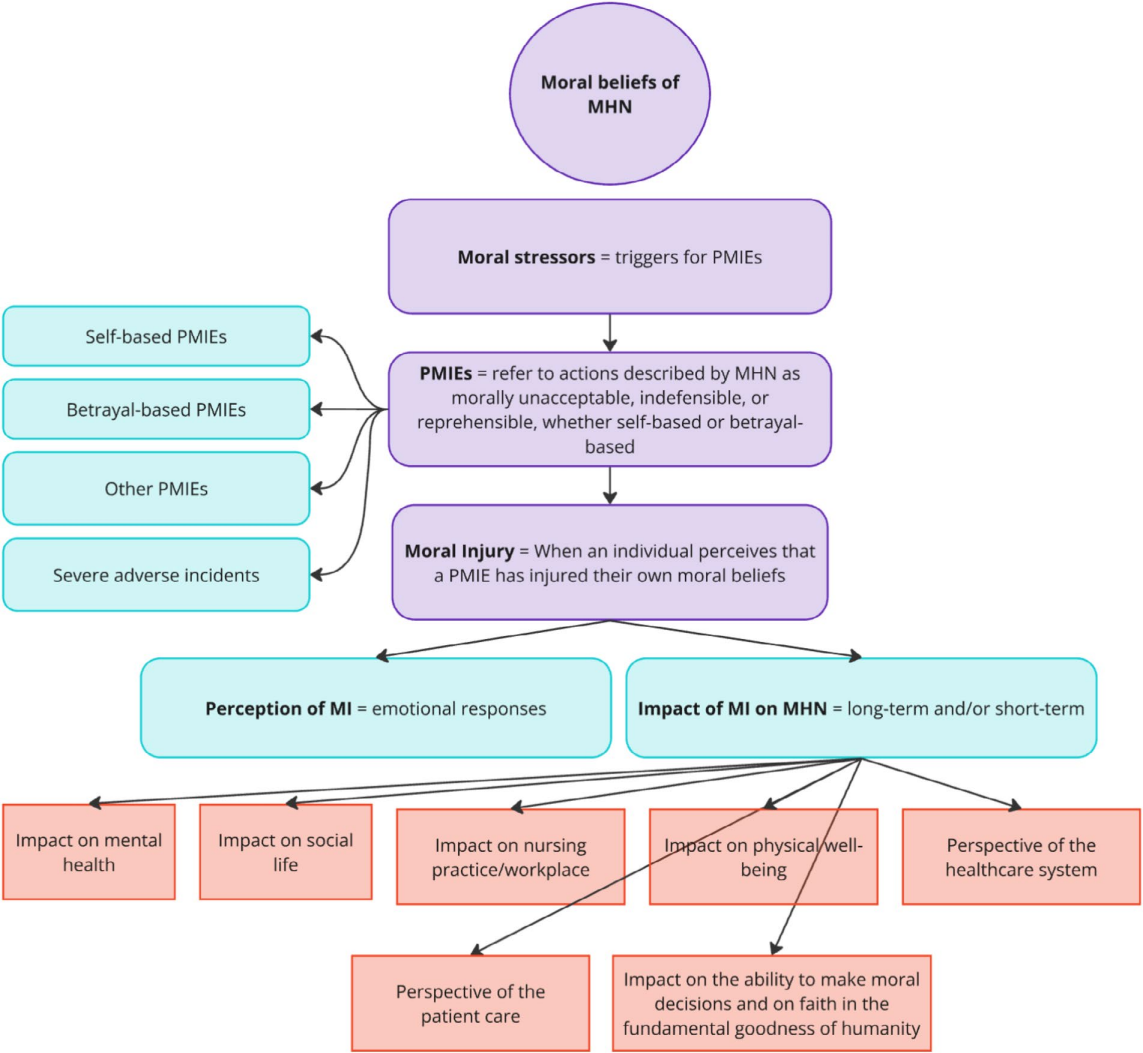
Main Categories (purple) and Subcategories (blue). *Source:* own illustration.

The analysis, conducted through a deductive approach for main categories and an inductive approach for subcategories, identified four main categories encompassing moral values/beliefs, moral stressors, PMIEs, and MI. Figure 1 highlights the main categories (purple). Interviewees reported a diverse range of moral stressors leading to PMIEs, manifesting as MI. The results follow a chronological order, mirroring the progression of MI, starting with moral beliefs as the foundation of MI. Moral stressors, identified in our study as potential triggers for PMIEs, were found to subsequently lead to MI.

### 3.1 Moral Beliefs of MHN

The questioned MHN prioritised moral beliefs and values like not harming, doing good, benefiting patients, respecting needs, and promoting autonomy. They also emphasised values like respect, honesty, justice, and empathy towards both patients and staff, integral to their professional commitment. They aspired to align their actions with their professional ethos and aimed to ensure the provision of morally and ethically justifiable care. As they aspired to align their actions with their professional ethos and aimed to ensure the provision of morally justifiable care, it became evident that any deviation from these principles could lead to significant moral distress. This was particularly apparent in the analysis of various domains identified as moral stressors, which revealed patterns and trends that may have led to PMIEs and, consequently, to MI.

That's also something that violates my morals somehow. Because I actually want to do equal justice to all patients, in terms of ethical principles, justice. (PCI1)

### 3.2 Moral Stressors

The analysis of various domains identified as moral stressors shows patterns and trends that may lead to PMIEs and, consequently, to MI. The most frequently identified causes of PMIEs were moral stressors generated within the work environment, often due to a participant's lack of knowledge and professional experience, particularly in dealing with aggression incidents and employing appropriate techniques for it. This led to participants finding themselves in situations or immersing themselves in scenarios that were potentially morally injurious. According to interviewees, this contributed to overwhelm and stress, with increased workload and staffing shortages exacerbating these conditions and leading to PMIEs in the longer term.

And this [regarding the prevention of coercive measures] is sometimes not possible due to staff shortages. In acute wards, with inexperienced staff. Due to, insufficient personnel, due to deployments by individuals who have no idea about psychiatry. (PCI8)

Interviewees described hierarchical structures or the feeling of not being heard that impeded making decisions in morally challenging situations. Participants said that different perceptions of acceptable behaviour among different professional groups reinforced the feeling of not being heard. The feeling of not being heard contributed to participants feeling pressured into situations or actions that do not align with their moral values. They also said that a lack of trust or ineffective communication within a team creates a morally challenging environment that promotes PMIEs.

I can say what I want anyway, it doesn't matter anyway. (PCI2)

According to the study participants, moral stressors generated within the working environment, including lack of resources, a shortage of nursing staff, inadequate support from leadership, and infrastructural requirements, which in turn? increased moral distress. Interviewees described legal aspects, such as the absence of a doctor's prescription for certain measures, and political aspects, such as bed occupancy policies, resulting in their being unable to act in line with their moral values. This prevented them from acting in accordance with their moral principles and violated their moral values.

We mentioned that we are at our limit, so we cannot continue working. The staffing level was calculated for 16 patients, not for 18. (PCI1)

### 3.3 Potentially Morally Injurious Events

PMIEs were identified as behaviours and actions that participants described as conflicting with their moral values due to being transgressive, leading to MI. The self‐based PMIEs most frequently mentioned by participants that led to MI included coercive measures such as forced medication and closed isolation, power games, punitive measures, restrictive and limiting behaviour towards patients, and the omission of important regulatory or health‐promoting social measures, such as open communication about moral conflicts and dilemmas. In addition, participants perceived pejorative communication and the stigmatisation of patients as conflicting with their values.

So, for example, that I judge or condemn the person in the conversation or the patient. Instead of the situation. (PCI6)

Interviewees described betrayal‐based PMIEs as a lack of support from leaders and physicians, coercive measures, and power plays. Lack of support from leaders, especially in situations that participants described as morally reprehensible, left participants with a feeling of isolation and abandonment and potentially led to MI.

… you have an assignment, you carry it out, you question something ethical, but you get no support from the superiors … (PCI17)

Coercive measures, including closed isolation, forced medication, or restrictive actions, were described as betrayal‐based when participants found themselves involuntarily or unintentionally involved in morally unjustifiable situations.

Participants explained that power plays, such as sanctioned behaviours towards patients, often inevitably led to coercive measures, as these actions tended to provoke rather than de‐escalate the patients. This resulted in aggressive incidents, implying the need for coercive measures. According to those interviewed, they observed MHN sanctioning patients by prohibiting them from smoking or not responding to the patient's wishes. For the interviewees, it was morally unacceptable that these power plays ultimately led to coercive measures in which they were involuntarily involved.

So, from what I've also observed, these are indeed power plays from the staff towards the patients, where it might escalate, making things worse, and you end up having to use coercive measures afterwards. People feel provoked, both staff and patients. (PCI14)

PMIEs that cannot be attributed to either betrayal or self‐based categories are rooted in the political realm. Financial aspects prioritised over the needs of patients, particularly in the Swiss healthcare system, are perceived by participants as a cause of MI. Interviewees described moral stressors as resource shortages and insufficient staffing, leading to high workload, time constraints, and inadequate capacities as the cause of their inability to provide appropriate and necessary medical interventions, and as a result, PMIES, which are perceived as a cause of MI.

I mean, you only look at where can I get the maximum profit of each patient. Whether this serves his actual problem or not plays less and less of a role. (PCI18)

Participants reported that severe adverse incidents such as suicide attempts, verbal threats, and violence caused intense emotional reactions such as feelings of guilt, fear, anger, and helplessness.

I was strangled by a patient. I struggled for a long time with um touching my neck. Even from trusted people. (PCI3)

### 3.4 Moral Injury

#### 3.4.1 Perception of Moral Injury: Emotional Symptoms

A central element of MI is their perception and description by interviewees. The emotional symptoms of MI encompass a range of emotions triggered by PMIEs and are considered manifestations of MI. It is important to note that the perception of MI can be an individual, variable, and subjective experience, affecting how the injury is experienced. According to participants, MI triggered a broad spectrum of emotional responses, including anger, resentment, shame, guilt, helplessness, powerlessness, disappointment, frustration, and resignation. These emotional symptoms provide insight into the presence and impact of MI.

… that I feel guilty for doing something that is not right. (PCI14)

Participants' perceptions of MI were underscored by expressions such as *“quite intensive,”* “that shocked me”, *“felt used”*, or *“felt raped”*. These expressions highlight that intense emotions are valid indicators of deep dissatisfaction and moral conflict. Participants' perceptions suggested that certain actions and situations challenged and undermined their moral principles and beliefs. Interviewees described situations in which they could not act in the best interests of the patient and had to deviate from what they considered to be right or good. The feeling of not being able to help people, along with the pain of having to observe something that went against their moral values, formed the basis for MI. Participants said they were often forced to act contrary to their professional ethos and expressed concern about being confronted with situations that violated their values.

… I felt used and violated in a way, somehow. (PCI13)

According to the interviewees, anger and resentment could be triggered by various PMIEs, such as interactions with colleagues, a lack of understanding within specific situations, or larger issues within the healthcare system. They expressed that feelings of shame may be linked to their behaviour or that of a team member and stated that feelings of helplessness arose when they felt abandoned or overwhelmed by a situation or action. As described by the participants, frustration and resignation were frequent responses to moral stressors such as staff shortages, infrastructural challenges, or overall working conditions which again highlights the connection between moral stressors and MIs.

#### 3.4.2 Impact of MI on MHN Well‐Being and Practice

The results show that MI can have a physical, social, and psychological impact on participants. Negative effects occur both in the short and long term and serve as potential indicators for the development of MI. Participants described physiological symptoms of MI, including elevated heart rate, back and jaw pain due to traumatic experiences, stress, and overall physical exhaustion. They mentioned that MI also led to changes in social lives, diminishing patience in social environments, and making social activities less enjoyable due to the stress experienced.

… on the one hand, the physical fatigue, but also the combination with what I would now call mental restlessness … creates a tension that is almost unbearable. (PCI18)

Participants in the study described that, on a psychological level, MI leads to increased stress, sleep disorders, internal conflicts, panic attacks and anxiety. They often found it challenging to distance themselves from work, as thoughts about work events spiralled in their minds, making it difficult to switch off. Some interviewees blamed themselves for certain events and felt personally responsible. The stress led to health issues for participants, including disrupted appetite and increased addictive substance consumption. The fear of re‐traumatisation was of great relevance to the participants. They emphasised that the fear that similar situations will occur again played a crucial role in the experience of MI, causing uncertainty about appropriate actions and self‐defence. Some interviewees indicated that they felt quickly triggered by similar situations, leading to spontaneous emotional outbursts. Some reported experiences of anxiety when confronted with situations involving aggression or shouting. In some cases, they sought professional help from psychologists or psychiatrists, especially when their sleep was no longer restful, and when the stress became overwhelming.

Panic attacks. So, when someone screamed. Or when aggression was involved. I got really anxious. (PCI10)

According to the participants, MI influenced how they perceived their work. They began scrutinising their professional activity and their place within the profession, expressing doubts about the future of the profession and its compatibility with their moral values. As our data revealed, MI can show itself in strong emotions and also in struggles with the self‐concept of the interviewees in their perspective of patient care and the healthcare system. The self‐concept refers to how individuals perceive their identity, including their values, beliefs, and their place in their professional roles. For example, one participant reacted to MI by questioning their role:

… what am I doing here? This isn't my job, so what's the crap? (PCI12)

Interviewees described how they were no longer able to carry out similar interventions due to previous experiences, finding themselves frightened and reactive. Another aspect was the fear of not knowing how to respond appropriately in similar situations. These fears were accompanied by thoughts such as “I don't know how I will behave in the next situation” or “What will happen next?”. Some participants quit their jobs due to their experiences. This entailed temporarily transitioning to alternative healthcare settings, such as shifting from acute inpatient environments to day clinics or taking an extended hiatus from psychiatric practice. Many cases also indicate that participants perceived a compromise in the quality of care due to MI. In the descriptions provided by interviewees, this was expressed through more condescending communication or reduced patience in dealing with patients. They described and perceived that the distress caused by MI led to more coercive and sanctioning measures being taken that have not been deemed necessary.

Which ultimately led me to turn my back on both acute wards on the one hand, but also on the clinic as such on the other. (PCI19)

#### 3.4.3 Impact of MI on Moral Decision‐Making and Faith in Humanity

The analysis shows that the effects of MI on the ability to make moral decisions are diverse. Ten participants stated they had no doubts, while three mentioned occasional or career‐spanning doubts during their professional careers. Those without doubts emphasise their ability to make morally sound decisions despite their experiences with MI. Some emphasise the importance of continuous questioning in moral decision‐making.

Some interviewees described positive beliefs towards humanity and trust in the fundamental goodness of humanity despite experiences with MI, while others experienced mixed feelings and observed changes in their worldview.

“So, it's become harder to believe that.” [In response to the loss of the faith in humanity due to the experience of MI] (PCI2)

#### 3.4.4 Coping Strategies, Supportive and/or Protective Factors

Some interviewees reported coping strategies for dealing with MI, including self‐reflection and targeted strategies. Interpersonal exchanges played a central role, involving discussions with friends and team members about moral topics. Participants emphasised the importance of support from leaders and supervision in complex patient situations. Debriefings after coercive measures allowed them to provide feedback and discuss morally injurious situations, promoting collective strength and enhancing emotional well‐being.

We were then able to discuss it, and I could present my arguments in that direction. The situation could be resolved to a certain extent. (PCI16)

Interviewees with several years of experience mentioned that conditions in mental health nursing have changed, reducing the risk of PMIEs and, consequently, MI. They highlighted positive changes such as targeted aggression management, new adult protection laws, and increased coaching support. Many viewed MI as a critical learning experience that positively impacted their personal and professional development, fostering a more mindful attitude towards recognising PMIEs and MI early. They also developed strategies to cope with MI and advocate for their moral values.

… the values and attitude can support you. And knowing yourself, dealing with yourself. And always questioning it. (PCI4)

## 4 Discussion

This study investigated MI in Swiss mental health nursing, revealing its complexity and impact on nurses' well‐being and professional practice. MI experiences often involve a complex interplay of moral stressors, including organisational, political, and interpersonal factors, as well as betrayal or self‐based PMIEs (Litz et al. 2009; Litz and Kerig 2019). The study showed that moral stressors like nursing shortages and hierarchical structures were often the basis for PMIEs becoming injurious. Participants emphasised the significance of moral values in mental health nursing, aligning with the International Council of Nurses (ICN) Code of Ethics, which underscores promoting health, preventing illness, restoring health, and alleviating suffering as ethical standards (International Council of Nurses 2001). Severe adverse incidents, including suicide attempts, violence, and verbal abuse towards MHN, evoked similar emotional responses among participants. They are considered PMIEs potentially leading to MI according to Looi et al. (2022). This was confirmed by our study. Specific PMIEs in the mental health setting, including coercive measures, power plays, and sanctioning behaviour, were particularly pronounced. These experiences often involved participants directly or were observed when carried out by colleagues, exacerbated by staffing shortages. In addition to staff shortages, participants' accounts suggest that institutional factors such as a lack of ethical leadership, insufficient policy guidance, and organisational tolerance of harmful practices also contributed to PMIEs. This underscores the need to understand MI not only as an individual psychological reaction, but as a phenomenon embedded within broader structural and ethical frameworks. Power plays are frequently aligned with coercive measures due to feelings of inadequacy, overwhelm, and inexperience. The perceptions of MI involved intense questioning of these actions. They described a broad range of emotions that showed to be symptoms of a MI such as anger, disappointment, frustration, and resignation, along with feelings of guilt, shame, helplessness, and powerlessness. In addition, they expressed doubts about their self‐concept, questioning the healthcare system including the patient care practices. The repercussions of MI extended beyond emotional distress, with participants reporting professional challenges, including doubts about their career trajectory and the quality of care. Long‐term consequences included increased job turnover, sleep disturbances, anxiety, panic attacks, impaired work performance, substance abuse, and potentially depression, PTSD, and burnout. These findings align with previous MI research, which identifies similar symptoms and health outcomes for HCWs (Bowers et al. 2006; Jinkerson 2016; Jovarauskaite et al. 2022; Looi et al. 2022; Mantri et al. 2020; Stovall et al. 2020; Wang et al. 2021; Williamson et al. 2020). Litz and Kerig (2019) posit that the risk of MI increases with the duration and frequency of exposure to moral stressors and that PMIEs become MI when they impact long‐term well‐being. However, it could be argued that this definition is too narrowly framed. Contrary to existing literature, this study defines MI as an event associated with negative emotional reactions and immediate impacts on the personal well‐being of MHN and the attitude towards their work, based on data collection and analysis. It is identified as an event associated with strong emotional reactions triggered by individual incidents, which participants vividly described. This decision is based on the subjective nature of this phenomenon, as participants' narratives highlight the immediacy and intensity of their emotional experiences. Therefore, exclusively defining MI by its long‐term effects is not justified due to the diversity in individual narratives. Acknowledging the varied emotional responses and interpretations of MI among individuals is crucial.

This study contributes to clarifying the connection between moral stressors, PMIEs, and MI. It contributes to understanding MI in psychiatric nursing in Switzerland and underscores the importance of addressing moral distress in healthcare settings. Notably, the COVID‐19 pandemic intensified research on MI, particularly in acute care settings among frontline nurses (Čartolovni et al. 2021; Maguen and Griffin 2022). Unlike these studies, which focus on the immediate impact of the pandemic, this research addresses MI within the context of psychiatric nursing in general. Veteran studies have traditionally framed MI within the context of betrayal‐based and self‐based PMIEs, emphasising prolonged exposure to moral stressors (Litz and Kerig 2019). In contrast, this study suggests that MI in psychiatric nursing can arise from specific, acute incidents, whether self‐ or betrayal‐based, as well as chronic systemic issues, highlighting the immediate and long‐term impacts on MHN.

As a qualitative descriptive study, the duration and frequency of exposure to moral stressors were not specified. Thus, contrary to Litz and Kerig (2019), no classification regarding the duration, frequency of events, and the extent of the damage caused by MI can be made. However, indications suggest participants faced prolonged moral stressors, like nursing shortages, leading to MI with long‐term impacts on personal well‐being and nursing practice.

A comparison with military and COVID‐19‐related MI research highlights cultural and systemic differences. While MI in military settings often involves prolonged exposure to traumatic events, the COVID‐19 pandemic intensified MI due to sudden, overwhelming stressors (Litz and Kerig 2019; Čartolovni et al. (2021); Maguen and Griffin 2022). In contrast, MI in Swiss mental health nursing is influenced by systemic issues like staffing shortages and hierarchical structures, which may shape moral stressors differently. Exploring these contextual differences can help tailor interventions to specific settings.

Future research examining the long‐term effects of MI on psychopathological aspects, as well as studies on coping strategies and the prevalence of MI in psychiatric settings, would be warranted based on the present data. A longitudinal follow‐up of participants would provide valuable insights into whether MI symptoms persist, worsen, or improve over time, thereby advancing the understanding of the development and progression of MI in mental health nursing. Furthermore, future research should consider developing a classification framework for MI severity (e.g., mild vs. severe) to improve the practical applicability of the concept and facilitate the design of targeted interventions.

### 4.1 Limitations and Quality Criteria

As participants worked in two psychiatric clinics in German‐speaking Switzerland, the results might not represent the experiences of the broader mental health nursing workforce. Furthermore, the research focuses on MI in MHNs but lacks a comparison with other mental health professionals, such as psychiatrists and social workers. While such a comparison would broaden the perspective and highlight the uniqueness of MHNs' experiences, there is a lack of research comparing MI across different psychiatric professions. This emphasises the need for focused studies on MHNs, making this research particularly relevant. Differences in institutional culture, patient demographics, or available resources, such as those in rural settings, outpatient care, or private institutions, could influence the occurrence and impact of PMIEs. Therefore, generalisability beyond similar contexts should be considered with caution. However, the study illuminated the connections between moral stressors, PMIEs, and MI and contributed to the theoretical description of these concepts, thereby promoting transferability to other settings. To further enhance the transferability of the results, a detailed description of the study's procedures and the examined context was provided. The researcher acknowledges her background as an MHN, which could potentially introduce personal experiences and professional perspectives, leading to subconscious subjective biases. Reflexivity was maintained by explaining the potential influence of the researcher on the research process, accompanied by reflective discussions on personal knowledge and roles within the research process (Perkhofer et al. 2016). To minimise these influences, scientific analysis methods with objective standards were applied, and memos were documented during data collection to capture critical considerations. The authenticity of the study was achieved by illuminating the perceptions and descriptions of the participants. This involved utilising open‐ended questions during the interviews to elicit rich and detailed responses.

## 5 Conclusion

The findings suggest that MI can have an impact on nurses' well‐being in mental health nursing and their nursing practice. This implies that MI is not a concept solely relevant to the military context or the COVID‐19 pandemic. The present findings not only suggest that MHNs facing MI are at risk of negative health consequences such as anxiety and panic attacks, sleep disorders, and fear of re‐traumatisation, but also support the literature on veterans and COVID‐19, indicating potential similar long‐term effects in the realm of MHN. Given the results, it can be argued that the definition of MI should be more flexible to adequately consider an array of individual experiences including long‐ and short‐term consequences. It becomes evident that personal moral beliefs and boundaries play a crucial role in how MHNs interpret moral stressors and identify which PMIEs are morally injurious. This underscores and highlights the complexity associated with defining and capturing MI.

## 6 Relevance for Clinical Practice

The present study illuminates the complexity of MI in mental health nursing and underlines the need for interventions to prevent and support MHN in dealing with moral stressors, PMIEs, and MI. Practical strategies for preventing or mitigating the impacts of MI require commitment at organisational, management, and individual MHN levels. Organisations are responsible for proactively addressing moral stressors and preventing or mitigating these challenges to reduce MI and aim to strengthen staff well‐being. MHN benefits from having the opportunity to discuss difficult situations as part of debriefings and with their management, allowing them to cope and feel heard. Successful strategies for mitigating MI include ethical debriefings, Schwartz Rounds, and resilience training, which could help prevent MI or support affected staff. Additionally, adequate support following severe incidents is a necessity, inclusive of professional psychological support. Furthermore, the study highlights the influence of legal, political, and institutional factors on the experience of PMIEs and MI, emphasising their relevance to practice. Based on these findings, clinical managers, policymakers, and educators should develop context‐specific guidelines and training programmes to strengthen ethical practice environments and reduce the risk of MI in mental health nursing. This includes integrating MI awareness and ethical resilience training into mental health nursing education to better prepare MHNs for ethically challenging situations.

## Conflicts of Interest

The authors declare no conflicts of interest.

## Data Availability

The data that support the findings of this study are available on request from the corresponding author. The data are not publicly available due to privacy or ethical restrictions.
